# Supplementary figures and images for: Top-down control of sustained attention by the medial prefrontal cortex (mPFC) - locus coeruleus (LC) circuit during the rodent continuous performance test (rCPT)
Source: bioRxiv. 2025 Dec 12:2025.12.01.691673. Preprint. [Version 2] doi: 10.64898/2025.12.01.691673 (PMC12710799; doi:10.64898/2025.12.01.691673)

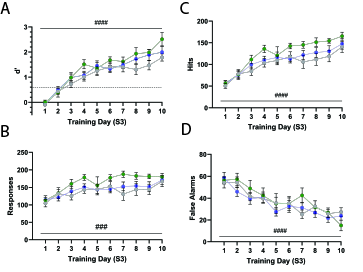

Supplement: Supplement 1 — Average performance by training day for each experimental group in mPFC-LCDREADDs manipulations as measured by d’ (A), total number of responses (B), number of hits (C), and number of false alarms (D)(hM3Dq: n=12, hM4Di: n=12, mCherry: n=8). PFCLChM3Dq mice are represented in green, mPFC-LChM4Di mice are represented in blue, and mPFCLCmCherry mice are represented in gray. All metrics were averaged by training day across stage 3 sessions; error bars represent SEM. #P < 0.05, ##P < 0.01, ###P < 0.001, ####P < 0.0001. [file media-1.tif]

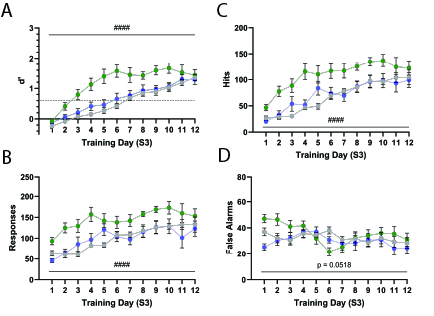

Supplement: Supplement 2 — Average performance by training day for each experimental group in mPFCDREADDs manipulations as measured by d’ (A), total number of responses (B), number of hits (C), and number of false alarms (D)(hM3Dq: n=10, hM4Di: n=10, mCherry: n=8). mPFChM3Dq mice are represented in green, mPFChM4Di mice are represented in blue, and mPFCmCherry mice are represented in gray. All metrics were averaged by training day across stage 3 sessions; error bars represent SEM. #P < 0.05, ##P < 0.01, ###P < 0.001, ####P < 0.0001. [file media-2.tif]

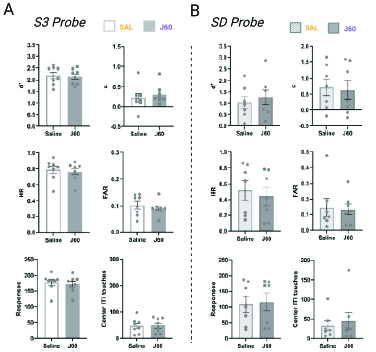

Supplement: Supplement 3 — A. Bar graphs showing comparative performance in rCPT behavioral metrics of mPFC-LCmCherry mice in SAL and J60 sessions during the S3 probe (n=8). B. Bar graphs showing comparative performance in rCPT behavioral metrics of mPFCLCmCherry mice in SAL and J60 sessions during the stimulus degradation (SD) probe (n=7). All metrics were averaged across same-treatment sessions; error bars represent SEM. *P < 0.05, **P < 0.01. [file media-3.tif]

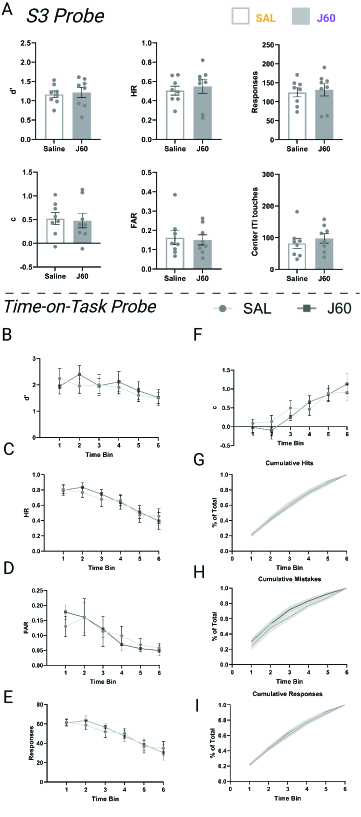

Supplement: Supplement 4 — A. Bar graphs showing comparative performance in rCPT behavioral metrics of mPFCmCherry mice in SAL and J60 sessions during the S3 probe (n=8). B-F. Line graphs showing comparative performance in rCPT behavioral metrics of mPFCmCherry mice by 15 min time bin in SAL and J60 sessions during extended sessions in the time-on-task (TOT) probe (n=8). All metrics were averaged across same-treatment sessions; error bars represent SEM. *P < 0.05, **P < 0.01. [file media-4.tif]

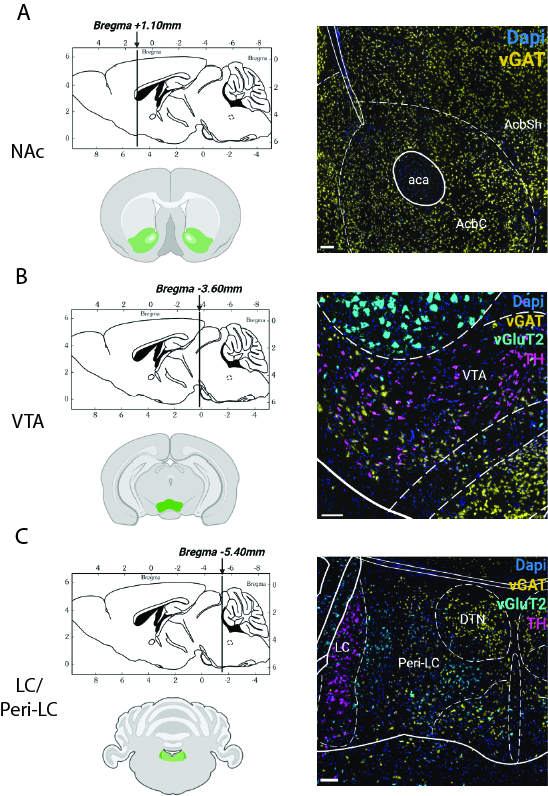

Supplement: Supplement 5 — Schematics and images showing the localization and alignment of slices in the NAc (A), VTA (B), and LC/Peri-LC region (C). Sagittal diagrams (top-left) show the coronal plane from which sections were taken. The regions analyzed are highlighted in green on schematics of coronal sections (bottom-left). Excitatory (vGluT2), inhibitory (vGAT), and catecholeminergic (Th) cell-type markers align with atlas-annotated topology of the respective regions. Scale bars = 200μm. [file media-5.tif]
